# A feature selection approach based on term distributions

**DOI:** 10.1186/s40064-016-1866-5

**Published:** 2016-03-01

**Authors:** Hongfang Zhou, Jie Guo, Yinghui Wang

**Affiliations:** School of Computer Science and Engineering, Xi’an University of Technology, Xi’an, 710048 Shaanxi China

**Keywords:** Feature selection, Term frequency, Term distributions, Text categorization

## Abstract

Feature selection has a direct impact on text categorization. Most existing algorithms are based on document level, and they haven’t considered the influence of term frequency on text categorization. Based on these, we put forward a feature selection approach, FSATD, based on term distributions in the paper. In our proposed algorithm, three critical factors which are term frequency, the inter-class distribution and the intra-class distribution of the terms are all considered synthetically. Finally, experiments are made with the help of kNN classifier. And the corresponding results on 20NewsGroup and SougouCS corpus show that FSATD algorithm achieves better performance than DF and *t*-Test algorithms.

## Background

With the rapid growth of network information, the number of electronic documents has drastically increased. The problem of how to organize these resources effectively has gained increasing attention from researchers (Shang et al. 2013). And text classification is the key to solve it. The goal of text categorization is to build a classifier based on some labeled documents and to classify the unlabeled documents into the prespecified categories (Yun et al. 2012). At present, text categorization has been widely applied in such fields as web documents categorization, information retrieval, E-mail filtering and Spam filtering. And many classification algorithms have been proposed, including decision tree (Quinlan 1986), support vector machine (SVM) (Cortes and Vapnik 1995) and k-Nearest Neighbors (kNN) (Yang and Pedersen 1997).

Decision tree uses the greedy strategies to construct an appropriate tree from a given training data set (Li et al. 2011). In a decision tree, each branch represents an outcome of the test on an internal node. Each leaf node denotes a class or a class distribution. A path traced from the root to a leaf node denotes a classification rule. In dealing with large and complex data sets, decision tree techniques are most widely used due to their high efficiencies. However, when there are large number of classes, the number of leaves becomes larger and it can cause the overlapping problem. In addition, errors can be accumulated and passed to deeper level. At last, it is difficult to design an optimal decision tree for classification.

Support vector machine is an effective technique to build classification models from high dimensional data. However, its computational complexity prohibits it from being used on very large training data. On the other hand, it is also difficult to build accurate models from data with a large number of classes. SVM methods map the data to another feature space by a kernel function so that a linear hyperplane can be found to separate the objects from different classes. It is hard for users to understand the internal details and working principles of the SVM classifier as SVM is like a black box.

kNN has been widely used in various types of classification tasks (He et al. 2003). This classification approach has gained its popularity owing to its low implementation cost and high effectiveness. However, kNN has a unique requirement which is the necessity in determining the appropriate value of parameter k.

At the moment, one of the difficulties in automatic text classification is “high dimensionality” property in feature space, which has reached up to tens or hundreds of thousands (Yang and Pedersen 1997). How to reduce the dimensionality of feature space and improve the efficiency and accuracy of classifiers become the most urgent problems to be solved in text categorization (Xu et al. 2008). So feature selection is a very critical step with a great influence on text categorization. And its task is to select the reasonable words, which have good abilities to distinguish categories from original feature space.

At present, some popular feature selection methods, such as document frequency (DF) and mutual information (MI) (Liu et al. 2014), are widely used in text categorization. These methods are all feasible in theorem, but their effects are different when they are applied in practices. All of these methods are compared by Shan et al. (2003). The experimental results show that DF has a low algorithmic complexity and it is easy to implement, but its performance is not ideal. And the performance of MI is the worst. It is not difficult to find that such methods almost use DF. In fact, term frequency also has a great influence on feature selection. So far, few effective methods have been proposed from the perspective of term frequency. Wang et al. (2014) proposed a *t*-Test feature selection approach based on term frequency, but it didn’t consider the interactions between categories sufficiently. In addition, n-gram (Liu and Lu 2007) is also used in text categorization and has achieved good results. While in training phase, n-gram always produces large amounts of noisy data which influences the training efficiency severly. And in testing phase, such noisy data also has a negative impact on accuracy. In view of these, we propose a new algorithm-FSATD (Feature Selection Approach based on Term Distributions), in which term frequency, the inter-class and the intra-class distribution of the terms are all considered synthetically.

The remaining of the paper is organized as follows: “Related works” section describes the related work about feature selection metrics, such as DF and *t*-Test. “FSATD” section proposes our new feature selection method-FSATD and gives a detail description about it. “Experiments setup” section describes the experimental data sets, document representation, classifiers, and performance measures used in our experiments. “Results and discussion” section presents the experimental results and shows the effectiveness of FSATD. Conclusion of the research is presented in “Conclusion” section.

## Related works

To deal with massive documents corpus, many feature selection approaches have been proposed. Through feature selection methods, we can select informative words, and then improve the classification accuracy. And its main idea is as follows. Firstly, it uses the feature selection function to compute some important values of each word in feature space. Secondly, it sorts the words in descending order according to above values. And finally, it selects the top N words to construct the feature vector. In this section, we only give definitions of two feature selection methods. And they are DF and *t*-Test respectively.

### Document frequency

Document frequency of a term is the number of documents which contain the term in the dataset. The term can be reserved only when it appears in adequate documents. To reduce the dimensionality of feature space and improve the classification accuracy, the terms whose DF is lower than a certain threshold will be removed from feature space (Xu et al. 2008).

Document frequency is a simple word reduction technology. Due to its linear complexity, it can be easily used in feature selection in face of large-scale corpus.

### *t*-Test

*t*-Test (Wang et al. 2014) is a feature selection approach based on term frequency, which is used to measure the diversity of the distributions of a term frequency between a specific category and the entire corpus. And it is defined as follows.

1 $$t\hbox{-}test(t_{i},C_{k})=\frac{|\overline{tf_{ki}}-\overline{tf_{i}}|}{\sqrt{\frac{1}{N_{k}}-\frac{1}{N}}*{s_{i}}}$$

Here, $\overline{tf_{ki}}$ is the average frequency of term $t_{i}$ within the category $C_{k}$, $\overline{tf_{i}}$ is the average frequency of term $t_{i}$ in collection *D*, $N_{k}$ is the document number in category $C_{k}$, *N* is the document number in collection *D*, ${s_{i}}^2=\frac{1}{N-K}{\sum \nolimits _{k=1,j\in {C_{k}}}^{K}(tf_{ij}-\overline{tf_{ki}})^2}$, and *K* is the category number in collection *D*.

The following two ways are used alternatively when the main features are finally selected.

2 $$t\hbox{-}test_{avg}(t_{i})= \sum \limits _{k=1}^{K}p(C_{k})*t\hbox{-}test(t_{i},C_{k})$$

3 $$t\hbox{-}test_{max}(t_{i})= \max \limits _{k=1}^{K}\{t\hbox{-}test(t_{i},C_{k})\}$$

Generally, the method shown in Eq. (2) is always better than that shown in Eq. (3) for multi-classes problem.

## FSATD

In this section, we propose a feature selection approach based on term distributions. The purpose of feature selection is to select the terms whose classification capabilities are stronger comparatively in feature space (Xu et al. 2008). In this algorithm, we measure the classification capability of the term based on the inter-class and intra-class distributions of terms.

### Variance

In the field of mathematical statistics, variance is usually used to measure the fluctuation of a set of data, and its value is positive correlated to the degree that a set of data deviates from the average. Its definition is as follows.

For a set of data $x_{1},x_{2},x_{3},\ldots ,x_{n}$ (*n* is the number of these data), $\bar{x}$ is the average of the set of data, which is shown as follows.

4 $$\bar{x}=\frac{1}{n}(x_{1}+x_{2}+x_{3}+\cdots +x_{n})$$

Then, the variance of the data set is $s^2=\frac{1}{n}[(x_{1}-\bar{x})^2+(x_{2}-\bar{x})^2+\cdots +(x_{n}-\bar{x})^2]$, and we can get Eq. (5) after simplifying.

5 $$s^2=\frac{1}{n}\sum \limits _{i=1}^{n}(x_{i}-\bar{x})^2$$

From Eq. (5), we can know that when the data distribution is scattered or the fluctuation of a data set is large, the sum of squared the difference between each data and the average is large. And it means the variance is large. Similarly, when the data distribution is centralized, the variance is small. So, the larger the variance is, the bigger the data fluctuation is. That is to say, the data is less stable. And likewise, the data set is stable when the variance is small.

In the paper, variance is used to select features in text classification.

1. Intra-class distribution of the term.

For a specific term $t_{i}$, $\{tf_{i1},\ldots ,tf_{ij},\ldots ,tf_{iN_{k}}\}$ is used to express the term frequency in every document within category $C_{k}$. Here, $N_{k}$ is the number of documents in category $C_{k}$, and $tf_{ij}$ is the term frequency of $t_{i}$ in document $d_{j}$. When the variance of $\{tf_{i1},\ldots ,tf_{ij},\ldots ,tf_{iN_{k}}\}$ is small, the fluctuation will be small. And it means the distribution of term $t_{i}$ in category $C_{k}$ is homogeneous. So the classification capability of term $t_{i}$ is strong.

2. Inter-class distribution of the term.

For a specific term $t_{i}$, $\{\overline{tf_{1i}},\ldots ,\overline{tf_{ki}},\ldots ,\overline{tf_{Ki}}\}$ is used to express the average frequency in every category. And here, *K* is the number of categories in collection *D*, and $\overline{tf_{ki}}$ is the average frequency of $t_{i}$ within a single category $C_{k}$. The larger the variance of $\{\overline{tf_{1i}},\ldots ,\overline{tf_{ki}},\ldots ,\overline{tf_{Ki}}\}$ is, the larger the fluctuation will be. This shows that the inter-class distribution of $t_{i}$ is uneven and the classification capability of term $t_{i}$ is strong.

### Term distribution

In this section, feature selection function is constructed based on variance. And some symbols are introduced firstly.

$tf_{ij}$ is the times that the term $t_{i}$ appears in document $d_{j}$, namely, term frequency.

$\overline{tf_{ki}}$ is the average frequency of term $t_{i}$ within the single category $C_{k}$. The formula is as follows.

6 $$\overline{tf_{ki}}=\sum \limits _{j=1}^{N}tf_{ij}\cdot {I(d_{j},C_{k})/N_{k}}$$

where *N* is the number of documents in collection *D*, $N_{k}$ is the number of documents in category $C_{k}$, and $I(d_{j},C_{k})$ is an indicator to discriminate whether document $d_{j}$ belongs to category $C_{k}$.

$\overline{tf_{i}}$ is the average term frequency of term $t_{i}$ in collection *D*, and it is calculated as follows.

7 $$\overline{tf_{i}}=\frac{1}{N}(tf_{i1}+tf_{i2}+\cdots +tf_{iN})$$

Similarly, *N* is the number of documents in collection *D*.

According to the definition of variance, we can construct the feature selection function from the following two aspects.

1. Intra-class distribution of the term.

Generally speaking, the term which has a good ability to distinguish category should have a high term frequency in the category, and the intra-class distribution of the term should be homogeneous. If a term $t_{i}$ appears only in few documents within the single category $C_{k}$, $t_{i}$ will be hardly selected as main feature no matter how large the term frequency is. So, the more homogeneous the intra-class distribution of the term is, the stronger the classification capability of the term will be. Then we will get Eq. (8) to measure the classification capability of the term.

8 $${s(t_{ki})}^2=\frac{1}{|C_{k}|}\sum \limits _{j\in {C_{k}}}(tf_{ij}-\overline{tf_{ki}})^2$$

where $|C_{k}|$ is the number of documents in category $C_{k}$, $tf_{ij}$ is the term frequency of term $t_{i}$ in document $d_{j}$, and $\overline{tf_{ki}}$ is the average frequency of term $t_{i}$ within the single category $C_{k}$. It is easy to find when the variance of set $\{tf_{i1},\ldots ,tf_{ij},\ldots ,tf_{i|C_{k}|}\}$ is small, the fluctuation is small. And this means that the distribution of term $t_{i}$ in category $C_{k}$ is homogeneous. So the classification capability of term $t_{i}$ is strong.

2. Inter-class distribution of the term.

The inter-class distribution of the term also has an effect on the classification capability. If a term $t_{i}$ appears almost in every category, the classification capability of $t_{i}$ will be weak. And likewise, if a term $t_{i}$ appears only in one category and the distribution in the category is homogeneous, $t_{i}$ will have a good ability to distinguish categories. Hence, the less homogeneous the inter-class distribution of the term is, the stronger the classification capability of the term will be. So, we will get Eq. (9) as the following.

9 $${s(t_{i})}^2=\frac{1}{K}\sum \limits _{k=1}^{K}(\overline{tf_{ki}}-\overline{tf_{i}})^2$$

Here, *K* is the number of categories in collection *D*, $\overline{tf_{ki}}$ is the average frequency of term $t_{i}$ within the single category $C_{k}$, and $\overline{tf_{i}}$ is the average frequency of term $t_{i}$ in collection *D*. It’s easy to see when the variance of set $\{\overline{tf_{1i}},\ldots ,\overline{tf_{ki}},\ldots ,\overline{tf_{Ki}}\}$ is large, the fluctuation will be large. This reflects that the inter-class distribution of $t_{i}$ is uneven and the classification capability of term $t_{i}$ is strong.

According to these two points, it’s clear that the classification capability of the term is strong when the inter-class distribution of the term is uneven and the intra-class distribution of the term is homogeneous. Besides, term frequency of term $t_{i}$ also has an effect on the classification capability. It means the term which has a good ability to distinguish category should have a high term frequency in the category. So term frequency should be used to construct feature selection function. So we can get the following formula.

10 $$F(t_{ki})=\frac{{s(t_{i})}^2}{{s(t_{ki})}^2}*\frac{\overline{tf_{ki}}}{\overline{tf_{i}}}$$

Finally we construct the following function to measure the classification capability of the term.

11 $$G(t_{i})=\frac{1}{\lambda }*\sum \limits _{1\le {q}<r\le {K}}|F(t_{qi})-F(t_{ri})|$$

Here, $\lambda =\frac{K!}{(K-2)!*{2!}}$, and *K* is the number of categories in collection *D*. Experiment results show that features selected by the proposed approach have stronger abilities to classify texts.

### Algorithm description

According to above, we present a new feature selection algorithm, FSATD, based on the distributions of terms. Its pseudocode is as follows.

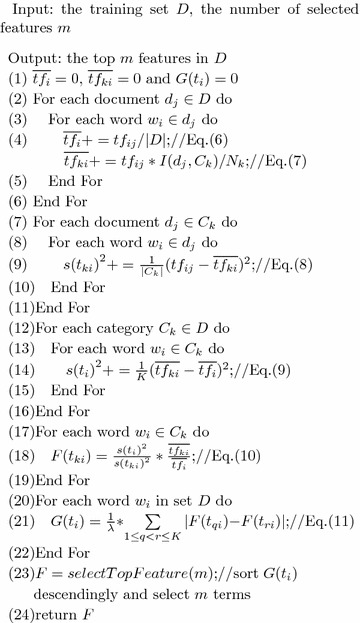



## Experiments setup

The experiments are performed on a PC with operating system of Windows 7, an i3 CPU (2.40GHz) and an 8G memory. The programming environment is JDK 1.6.

### Experimental data

In our experiments, we use the popular datasets-20NewsGroup and SougouCS.

The 20NewsGroup corpus is a collection of about 20,000 newsgroup documents nearly evenly distributed among 20 discussion groups, and every group consists of 1000 documents. All letters are converted into lowercase, and the word stemming is applied. In addition, we use the stop words list to filter words.

The corpus SougouCS is from Sogou Laboratory. As the number of web pages in some classes is too small, we only choose 12 classes. And they are car, finance, IT, health, sports, tourism, education, culture, military, housing, entertainment and fashion respectively.

### Document representation

Documents are represented by Vector Space Model (Zhang 2010; Salton et al. 1975). That is, the content of a document is represented by a vector in the term space. It is illustrated in details as the following. $V(d)=(t_{1},w_{1}(d);\ldots ; t_{i},w_{i}(d);\ldots ;t_{n},w_{n}(d))$, where *n* is the number of terms in a document *d*, and $w_{i}(d)$ is the weight of a term $t_{i}$ in document *d*. In experiments, TF-IDF (Term Frequency-Inverse DF) (Xiong et al. 2008; Salton and Buckley 1988) is used to calculate the weight.

### Classifier selection

In the experiments, kNN classifier (Chen 2011) is used as the basic classifier. kNN is widely used in text classification as it is easy and has lower error rate in relative terms. The similarity measure used for the classifier is the cosine function.

In kNN, training data set and testing data set are required. So we randomly select 67% instances from each category as training data and the rest as testing data (Wang et al. 2014).

### Performance measures

We measure the effectiveness of classifiers in terms of the combination of precision (*p*) and recall (*r*) which are widely used in text categorization. That is, we use the well-known $F_{1}$ function (Sebastiani 2002) as follows.

12 $$F_{1}=\frac{2*{p}*{r}}{p+r}$$

For multi-class text categorization, $F_{1}$ is usually estimated in two ways. And they are the macro-averaged $F_{1}$ (macro-$F_{1}$) and the micro-averaged $F_{1}$ (micro-$F_{1}$). In this paper, we only use macro-$F_{1}$, as shown in Eq. (13).

13 $$macro\hbox{-}F_{1}=\frac{ \sum \nolimits _{k=1}^{K}F_{1}(k)}{K}$$

where $F_{1}(k)$ is the $F_{1}$ value of the predicted *k*th category.

## Results and discussion

The kNN classifier is sensitive to the value of *k*. So we have a comparative study with the performance of FSATD, DF and *t*-Test on 20NewsGroup and SougouCS corpus with the different *k* values.

**Fig. 1:**
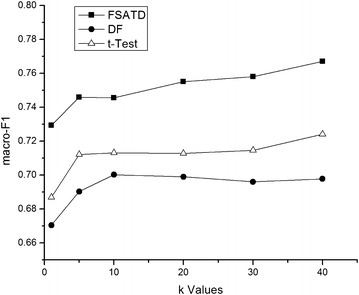
Macro-$F_{1}$ performance on the 20NewsGroup corpus with the different *k* values. The kNN classifier is sensitive to the value of *k*. So we have a comparative study with the performance of FSATD, DF and *t*-Test on 20NewsGroup corpus with the different *k* values. The classification results on 20NewsGroup corpus with the different *k* values are shown

**Fig. 2:**
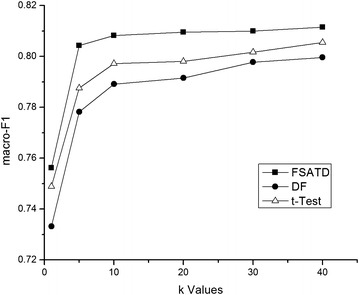
Macro-$F_{1}$ performance on the SougouCS corpus with the different *k* values. We have a comparative study with the performance of FSATD, DF and *t*-Test on SougouCS corpus with the different *k* values. The classification results on SougouCS corpus with the different *k* values are shown

The classification results on 20NewsGroup and SougouCS corpus with the different *k* values are shown in Figs. 1 and 2. The results show that the macro-$F_{1}$ values of FSATD, DF and *t*-Test are different with different *k* values. But FSATD consistently outperforms DF and *t*-Test in the performance of macro-$F_{1}$ values no mater what value *k* is. So we set $k=20$ in the follow-up experiments in view of the sizes of the two data sets and the classification performance.

**Fig. 3:**
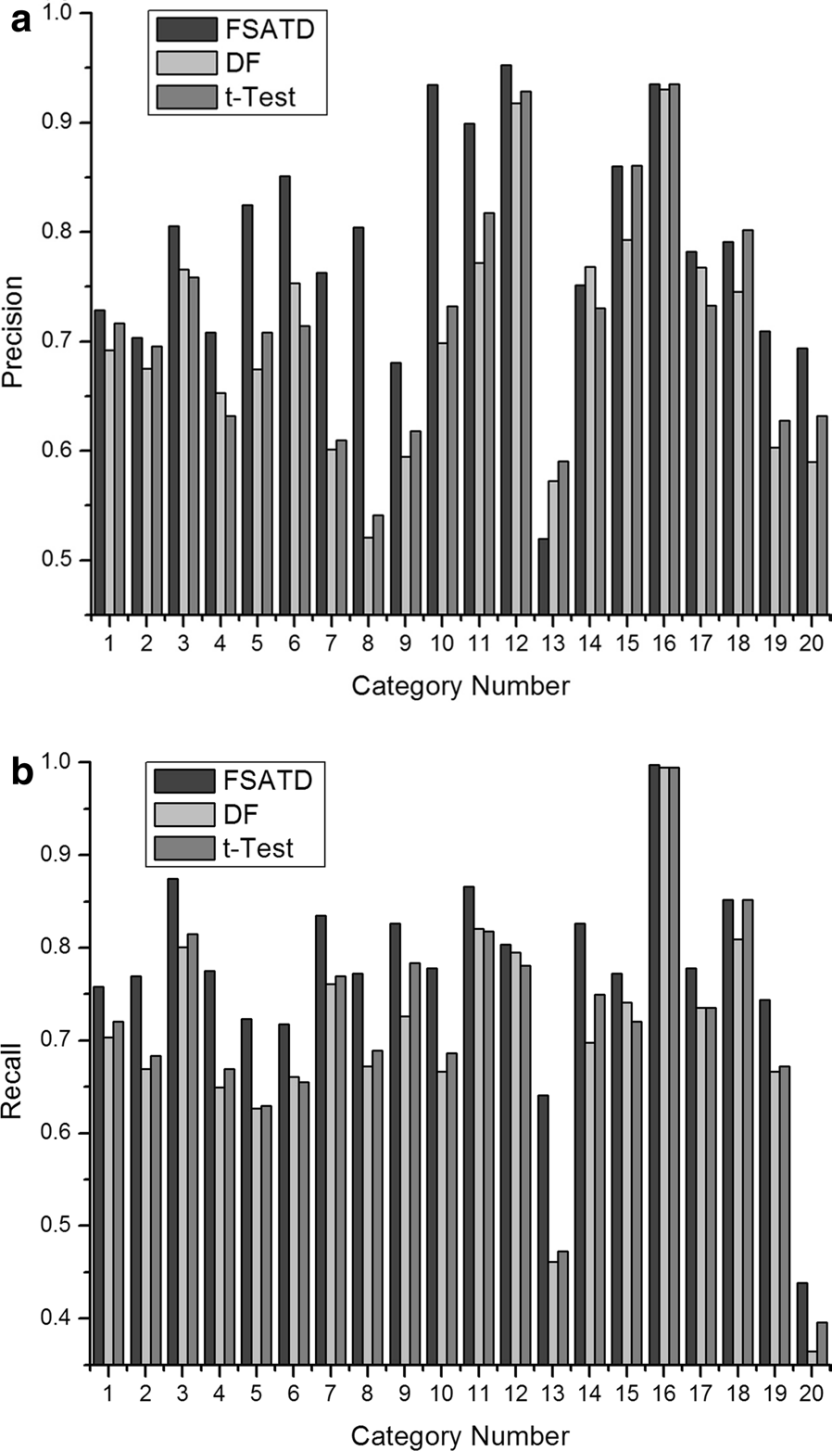
Precision and recall performance on the 20NewsGroup corpus. In our experiments, 1500 features are selected for convenience in feature space. And figure shows the precision and recall of DF, *t*-Test and FSATD on the 20NewsGroup corpus. **a** Precision. **b** Recall

Figure 3 shows the precision and recall of DF, *t*-Test and FSATD on the 20NewsGroup corpus. And in our experiments, 1,500 features are selected for convenience in feature space. It is clear that FSATD achieves better performance than DF and *t*-Test, and the precision and recall of most categories have some improvements.

In Fig. 3, the corresponding relationships are given between the category number and the actual category. 1-alt.atheism, 2-comp.graphics, 3-comp.os.ms-windows.misc, 4-comp.sys.ibm.pc.hardware, 5-comp.s-ys.mac.hardware, 6-comp.windows.x, 7-misc.forsale, 8-rec.autos, 9-rec.motorcycles, 10-rec.sport.baseball, 11-rec.sport.hockey, 12-sci.crypt, 13-sci.electronics, 14-sci.med, 15-sci.space, 16-soc.religion.christian, 17-talk-politics.guns, 18-talk.politics.mideast, 19-talk.politics-misc, 20-talk.religion.misc.

**Fig. 4:**
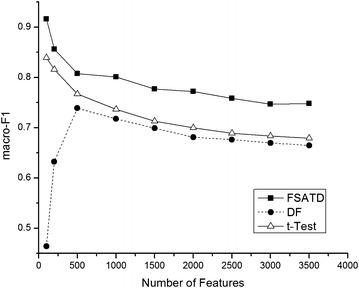
Macro-$F_{1}$ performance on the 20NewsGroup corpus. In order to verify the performance of FSATD on the 20NewsGroup corpus, different dimensionalities are selected when the dimensionality of feature space varies. And finally we compare their values of macro-$F_{1}$ for three algorithms. And the details are shown

In order to verify the performance of FSATD on the 20NewsGroup corpus, different dimensionalities are selected when the dimensionality of feature space varies. And finally we compare their values of macro-$F_{1}$ for three algorithms. And the details are as follows (Fig. 4).

As shown, it is clear that FSATD achieves better performance than DF and *t*-Test ones. When the dimensionality of feature space is reduced, their differences show to be bigger among three algorithms.

**Fig. 5:**
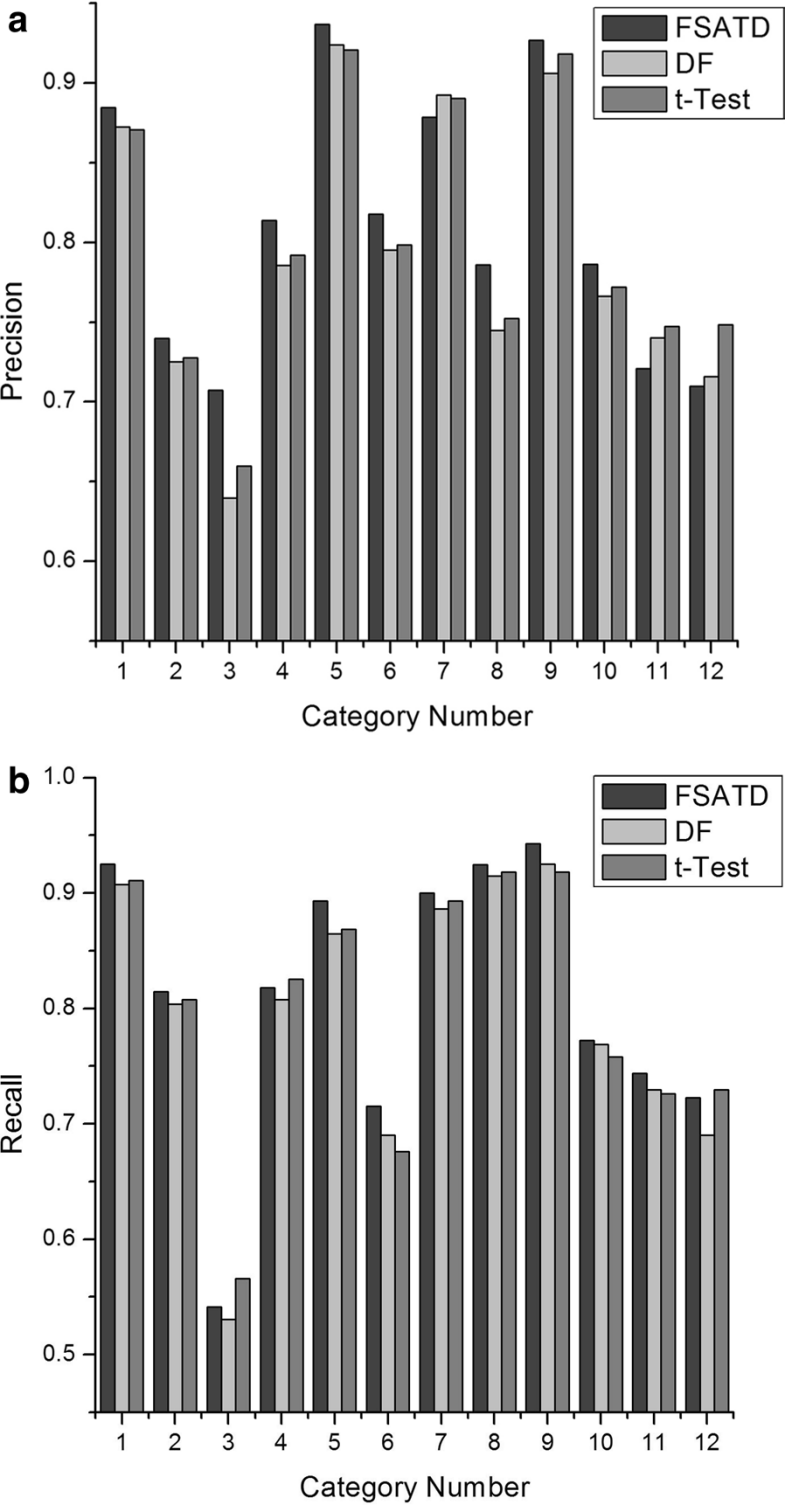
Precision and recall performance on the SougouCS corpus. In our experiments, 4500 features are selected in feature space. And figure shows the precision and recall of DF, *t*-Test and FSATD on the SougouCS corpus. **a** Precision. **b** Recall

Figure 5 depicts the precision and recall performance of DF, *t*-Test and FSATD on the SougouCS corpus when 4500 features are selected in original feature space. It is clear that FSATD achieves better performance than DF and *t*-Test in most categories. But for a few categories, FSATD does not get better precisions. Through analyzing, we find that some categories, such as fashion and entertainment, have many common words which make the boundaries between categories obscure and have a negative impact on precision. In these categories, the intra-class distributions of these words are uneven, and the number of documents which contain the common words is low. DF selects features according to the DF. The word can be reserved only when it appears in adequate documents. As the DF of the common words is low, DF is not easy to select them as their main features. During selecting features, *t*-Test mainly considers intra-class distributions of the words. However, the intra-class distributions of the common words are uneven, so *t*-Test also does not readily select them as main features. FSATD considers the inter-class and the intra-class distributions of the words sufficiently. While these common words appear only in a few categories, so the value of the words calculated by FSATD is high. Therefore in these categories, FSATD is inclined to select the common words as features compared to DF and *t*-Test.

In Fig. 5, the category numbers represent the categories respectively as follows. 1-car, 2-finance, 3-culture, 4-health, 5-housing, 6-IT, 7-education, 8-military, 9-sports, 10-tourism, 11-fashion, 12-entertain-ment.

**Fig. 6:**
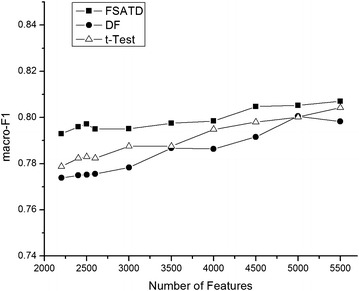
Macro-$F_{1}$ performance on the SougouCS corpus. Figure depicts the macro-$F_{1}$ performance of the three algorithms on the SougouCS corpus and different dimensionalities are selected when the dimensionality of feature space varies. We compare their values of macro-$F_{1}$ for three algorithms. And the details are shown

Figure 6 depicts the macro-$F_{1}$ performance of the three algorithms on the SougouCS corpus, which has the similar result to Fig. 4.

Through the above experiments, it is clear that FSATD achieves better performance than DF and *t*-Test. This is because term frequency, the inter-class and the intra-class distribution of the terms are all considered synthetically in FSATD. While DF only considers DF and *t*-Test mainly considers intra-class distribution of the word. So FSATD can select more reasonable features which have a positive impact on the classification performance than DF and *t*-Test.

## Conclusion

Feature selection plays an important role in text classification and has an immediate impact on text categorization. Most existing feature selection methods use DF. Through the analysis, we discover that term frequency has a great influence on feature selection. In view of this, we propose a feature selection approach based on term distributions in this paper. Additionally, term frequency is considered sufficiently. The experimental results on 20NewsGroup and SougouCS corpus show that FSATD achieves better performance than DF and *t*-Test.
